# Detection and Quantification Methods for Viable but Non-culturable (VBNC) Cells in Process Wash Water of Fresh-Cut Produce: Industrial Validation

**DOI:** 10.3389/fmicb.2020.00673

**Published:** 2020-05-04

**Authors:** Pilar Truchado, Maria I. Gil, Mar Larrosa, Ana Allende

**Affiliations:** ^1^Research Group on Quality, Safety and Bioactivity of Plant Foods, The Centre of Edafology and Applied Biology of Segura, Spanish National Research Council (CEBAS-CSIC), Murcia, Spain; ^2^Faculty of Biomedical and Health Sciences, Nutrition, Microbiota and Health Group, Universidad Europea de Madrid, Villaviciosa de Odón, Madrid, Spain

**Keywords:** flow cytometry, quantitative PCR, microbial inactivation, food safety, *Listeria monocytogenes*

## Abstract

The significance of viable but non-culturable (VBNC) cells in the food industry is not well known, mainly because of the lack of suitable detection methodologies to distinguish them from dead cells. The study aimed at the selection of the method to differentiate dead and VBNC cells of *Listeria monocytogenes* in process wash water (PWW) from the fruit and vegetable industry. Different methodologies were examined including (i) flow cytometry, (ii) viability quantitative polymerase chain reaction (v-qPCR) using an improved version of the propidium monoazide (PMAxx) dye as DNA amplificatory inhibitor, and (iii) v-qPCR combining ethidium monoazide (EMA) and PMAxx. The results showed that the flow cytometry, although previously recommended, was not a suitable methodology to differentiate between dead and VBNC cells in PWW, probably because of the complex composition of the water, causing interferences and leading to an overestimation of the dead cells. Based on results obtained, the v-qPCR combined with EMA and PMAxx was the most suitable technique for the detection and quantification of VBNC cells in PWW. Concentrations of 10 μM EMA and 75 μM PMAxx incubated at 40°C for 40 min followed by a 15-min light exposure inhibited most of the qPCR amplification from dead cells. For the first time, this methodology was validated in an industrial processing line for shredded lettuce washed with chlorine (10 mg/L). The analysis of PWW samples allowed the differentiation of dead and VBNC cells. Therefore, this method can be considered as a rapid and reliable one recommended for the detection of VBNC cells in complex water matrixes such as those of the food industry. However, the complete discrimination of dead and VBNC cells was not achieved, which led to a slight overestimation of the percentage of VBNC cells in PWW, mostly, due to the complex composition of this type of water. More studies are needed to determine the significance of VBNC cells in case of potential cross-contamination of fresh produce during washing.

## Introduction

Several studies have evidenced that many bacterial species, including foodborne pathogenic bacteria, develop stress resistance mechanisms that enable them to enter into a temporary state of low metabolic activity (Oliver, 2010). Under these conditions, cells can persist for extended periods without division, called dormancy or a viable but non-culturable (VBNC) state. The VBNC state has been defined as a survival strategy, where bacteria cannot grow on routine culture media but are alive and capable of renewing metabolic activity (Zhao et al., 2017; Dong et al., 2020). Recently, Dong et al. (2020) demonstrated that cells in the VBNC state go through different changes, including morphological and compositional variations, which allow them to have a higher resistance to chemical and physical stresses. Viable but non-culturable cells maintain their intact cell membrane and are metabolically active, to continue gene expression, having the ability to become culturable once resuscitated (Fida et al., 2017; Su et al., 2019). The occurrence of VBNC bacterial pathogens in food has been identified as a public risk concern (Makino et al., 2000). The presence of VBNC enterohemorrhagic *Escherichia coli* in salmon has been liked to a food poisoning incident (Dong et al., 2020). However, the significance of VBNC cells in the food industry related to cross-contamination during processing has not been elucidated, mostly because the available methodologies cannot differentiate dead and VBNC cells correctly in different matrixes. Therefore, there is a need to optimize the detection and quantification methods in different matrixes. Process wash water (PWW) has been recognized as one of the relevant vectors of microbial cross-contamination in the agro-food industries (Gil and Allende, 2018). Cross-contamination occurs when a contaminated product is washed and the pathogens are transferred from the contaminated product to the water and from the water to the clean product. Sanitizers are needed to maintain the microbiological quality of PWW, avoiding cross-contamination (Gombas et al., 2017). Chlorine is the most common sanitizer in the fresh produce industry. Generally, the efficacy of the sanitizers has been evaluated using plate counts (López-Gálvez et al., 2019). However, recently, Highmore et al. (2018) have demonstrated that chlorine induces the VBNC state of the foodborne pathogens *Listeria monocytogenes* and *Salmonella enterica*. Optimized quantification methods are required to determine the significance of the presence of VBNC cells in PWW.

The most popular methods to determine the presence and concentration of VBNC cells are staining techniques (Zhao et al., 2017). These techniques are based on the cell membrane integrity to differentiate between dead and VBNC cells, assuming that dead cells have the membrane damaged while VBNC and viable cells have an intact membrane (Oliver, 2010). However, as not all the dead cells have their cell membrane compromised, these methods can lead to an overestimation in the number of VBNC cells. The combination of dyes and flow cytometry has been widely used to determine the cell viability of foodborne pathogenic bacteria (Léonard et al., 2016), but it is not suitable for all the matrixes. Instead, viability quantitative polymerase chain reaction (v-qPCR) has been widely adopted to detect and quantify the presence of viable bacteria in specific food matrixes and water (Truchado et al., 2016; Dorn-In et al., 2019). The quantitative real-time PCR (qPCR) methodology has been combined with the use of photoreactive dyes such as propidium monoazide (PMA) and ethidium monoazide (EMA), as PMA-qPCR in food matrix and water (Codony et al., 2015). This technique is based on the ability of PMA to penetrate the dead cells which compromised membrane integrity and bind covalently to the DNA and free DNA after photoactivation, thus preventing subsequent PCR amplification (Nocker et al., 2006). On the other hand, EMA can diffuse across cell membranes using efflux pumps (Codony et al., 2015). However, these methodologies have to be validated for each type of matrix to avoid overestimation of the VBNC cells due to the presence of dead cells with an intact membrane (Nocker and Camper, 2009).

The objective of the present study was to optimize a suitable detection and quantification method of VBNC cells in PWW and the food safety significance for the agro-food industry. Different methodologies were examined, including (i) combination of dyes and flow cytometry; (ii) v-qPCR using PMAxx, an improved version of the PMA dye; and (iii) v-qPCR combining EMA and PMAxx. Additionally, the selected methodology was validated for the first time under industrial settings for PWW treated with chlorine. These studies were performed using *L. monocytogenes* as a model foodborne pathogen, which has been described to enter into the VBNC state (Highmore et al., 2018) and linked to listeriosis outbreaks in fresh produce (European Food Safety Authority [EFSA], 2018).

## Materials and Methods

### Bacterial Strains and Cocktail Preparation

For the inoculation of PWW, a six-strain cocktail of *L. monocytogenes* was used in this study. Strains were isolated from leafy vegetables, as previously described (Truchado et al., 2020). The *L. monocytogenes* strains were reconstituted in Brain Heart Infusion (BHI) broth (Oxoid, Basingstoke, United Kingdom) and consecutively subcultured twice in 10 ml of BHI, the first time at 37°C for 24 h and the second time at 37°C for 16 h. After the second incubation, 1 ml of each strain was combined to obtain a six-strain cocktail of *L. monocytogenes* (10^9^ cfu/ml).

To assay the suitability of detection methods to differentiate dead and VBNC cells, bacterial suspensions of dead and viable cells (10^9^ cfu/ml) were prepared as follows:

1. Heat treatment: The *L. monocytogenes* cocktail was exposed at 85°C for 20 min using a laboratory standard heat block.
2. Sanitizing treatment: Sodium hypochlorite was added to the six-strain cocktail of *L. monocytogenes* until a residual of 10 mg/l of free chlorine was reached to guarantee the complete inactivation of the cells. After a 1-min exposure time, 0.3 M of sodium thiosulfate pentahydrate (Scharlau, Barcelona, Spain) was added to quench the residual chlorine. Free chlorine concentration was measured with a digital chlorine colorimeter kit (DPD method; LaMotte model DC 1100, Chestertown, MD).

The cell inactivation after the treatments was confirmed by plating serial suspension dilutions of the treated *L. monocytogenes* cocktail in buffered peptone water (BPW, 2 g/l; Oxoid, Basingstoke, United Kingdom) on Oxford agar (Scharlau, Barcelona, Spain) followed by incubation at 37°C for 24 h. To inoculate PWW, the initial six-strain cocktail of *L. monocytogenes* (10^9^ cfu/ml) was centrifuged at 2,500 *g* for 5 min, and the supernatant was eliminated. The obtained pellet was washed twice with 18 ml of phosphate-buffered saline (PBS; Scharlau, Barcelona, Spain) using the same conditions as above. The cocktail was added to PWW to reach the desired concentration of approximately 10^5^ cfu/ml. The final concentration of the inoculum in the PWW was confirmed by plating duplicate serial suspension dilutions on ALOA/OCLA agar (Scharlau, Barcelona, Spain) followed by incubation at 37°C for 24 h.

### PWW Generated at Laboratory Scale

Process wash water from washing shredded lettuce was generated in the laboratory, mimicking the industrial conditions previously described (Tudela et al., 2019). Organic matter was measured as chemical oxygen demand (COD) determined by the standard photometric method (APHA, 1998) using the Spectroquant NOVA 60 photometer. The COD of the PWW was 1,700 mg/l.

### Live/Dead Flow Cytometry Analysis

Cell viability was determined by flow cytometry (LSRFortessa X-20 system) using the Live/Dead BacLight^®^ bacterial viability kit (Invitrogen, Waltham, United States) that contains two nucleic acid stains with different abilities to penetrate the bacterial cells: SYTO 9 and propidium iodine (PI). SYTO 9 is a cell-permeant green fluorescent dye that enters both live and dead cells. Propidium iodine is a membrane-impermeant dye that penetrates only in damaged or dead cells and emits red fluorescence upon intercalation with double-stranded DNA. When both dyes are used simultaneously, SYTO 9 is replaced by PI due to its higher affinity to bind DNA, quenching SYTO 9 fluorescence signal (Stiefel et al., 2015). As a result, red signals from cells are considered as “dead,” green signals as “alive,” and the double-staining cells as an intermediate state of membrane-compromised cells. The staining procedure was performed according to the manufacturer’s instructions.

Flow cytometry was used for measuring the viability of *L. monocytogenes* cells inoculated in PWW after treatment with chlorine. Untreated inoculated samples of PWW (50 ml) were used as controls and compared with inoculated PWW treated with 10 mg/l of free chlorine. This treatment reproduces the conditions found in industrial washing tanks. One milliliter of each PWW was mixed with 3 μl of both dyes (5 μM SYTO 9 and 30 μM PI) incubated for 15 min at room temperature in the dark. The green fluorescence emission of live bacteria was detected in the cytometer at 520 nm (FL1 channel), while the red fluorescence emission of compromised (double-stained) or dead bacteria was detected at 630 nm (FL4 channel). During data acquisition, all parameters were collected in the log mode, and data analysis was performed with the EC800 software version 1.3.6. (Sony Biotechnology Inc., Champaign, IL, United States). Forward and side scatter gates were established to exclude debris. Unstained and stained untreated live *L. monocytogenes* and isopropanol 70% dead *L. monocytogenes* were used as controls for gating the different regions and fluorescence adjustment.

### PMAxx v-qPCR

PMAxx (Biotium, Hayward, CA, United States), an improved version of the PMA dye for the selective detection of live bacteria by qPCR, was used as follows. PMAxx was diluted in sterile water to obtain a 2 mM stock solution and stored at -20°C in the dark until used. Separate flasks of PWW (50 ml) were inoculated with a cocktail of *L. monocytogenes* containing live (exponential phase), heat-treated, and chlorine-treated cells to approximately 10^4^ cfu/ml. From each flask, 10 ml of PWW was centrifuged at 4,000 rpm for 10 min at 4°C. The supernatant was removed, and the cell pellets resuspended in PBS at a final volume of 1,000 μl supplemented with PMAxx to obtain a final dye concentration of 50, 75, and 100 μM. After PMAxx addition, the samples were incubated at 200 rpm in the dark at room temperature or 40°C for 10–60 min. Stained samples were subsequently exposed to blue-light PMA-Lite LED photolysis (Interchim, Montluçon, France) for 15 min. In parallel, 10 ml of inoculated PWW was taken from each flask to determine the level of total bacteria by qPCR. Bacteria cells were concentrated by centrifugation (4,000 rpm, 4°C, 10 min). The supernatant was discarded, and untreated and PMAxx-treated pellets were kept at -20°C until DNA genomic extraction.

### EMA + PMAxx v-qPCR

Ethidium monoazide (Biotium, Hayward, CA, United States) was dissolved in sterile water to obtain 2 mM stock solution and stored at -20°C in the dark until used. Following the same procedure as previously described, three separate flasks of PWW (50 ml) were inoculated with a *L. monocytogenes* cocktail of live, heat-treated, and chlorine-treated cells. Ten milliliters from each flask was centrifuged, the supernatants discarded, and the pellets treated with a combination of 10 μM EMA and 75 μM PMA, incubated at room temperature and 40°C and activated with blue-light PMA-Lite LED photolysis as previously described. The total bacteria were also determined by qPCR, as described above.

### Culturable Bacteria

The concentration of *L. monocytogenes* (cfu/ml) in each of the different assays was confirmed by plating. Serial 10-fold dilutions were made in BPW (2 g/l; Oxoid) and plated in Oxford agar (Scharlau). Colonies were counted after 24 h of incubation at 37°C.

### PWW From an Industrial Setting

Samples of PWW were taken in an industrial processing line (Cuenca, Spain) of shredded iceberg lettuce washed in a large washing tank containing about 3,000 l of PWW. A residual concentration of 10 mg/l of chlorine was set to maintain the microbiological quality of the water. The washing was performed by immersion of cut lettuce for 30 s in the chlorinated water followed by a shower rinse with tap water for 30 s. The physicochemical and microbiological characteristics of the PWW were monitored each hour over 4 h (Table 1). The physicochemical analysis included pH, organic matter measured as COD, temperature, and oxidation-reduction potential (ORP) as well as free chlorine measurements determined as previously described (Tudela et al., 2019).

**TABLE 1:** Physicochemical and microbiological characteristics of the process wash water (PWW) at different sampling times including free chlorine (10 mg/l), pH, chemical oxygen demand (COD), temperature (T^a^), oxidation-reduction potential (ORP), and total counts (log cfu/100 ml).

Sampling time	Free chlorine (mg/l)	pH	COD (mg/l)	T^a^	ORP	Total count (log cfu/100 ml)
8:00	12.6	6.6	537 ± 11	4.0	818	2.4
10:00	14.6	6.5	503 ± 10	3.8	821	2.3
10:30	10.9	6.6	382 ± 20	3.8	807	2.6
11:00	9.0	6.5	469 ± 19	4.3	783	2.5

For microbiological analyses, three water samples (1 l each) per sampling time were taken. Samples were collected and processed, and levels of culturable bacterial populations enumerated as previously described (Tudela et al., 2019). For viable total bacteria, qPCR of EMA + PMAxx-treated samples was performed following the protocol previously described. Water samples (200 ml each) were vacuum filtered through sterile cellulose nitrate filters (0.45 μm). Filters were placed in falcon tubes (50 ml) containing 20 ml of PBS + Tween 80 (1 ml/l; Sigma-Aldrich, Saint Louis, United States) and shaken in a vortex for 2 min. After that, the filters were discarded and the tubes centrifuged for 10 min at 4,000 rpm. Then, the pellet was resuspended in sterile distilled water (1 ml) and transferred to clear transparent microtubes (2 ml). Then, the v-qPCR procedure was carried out as previously described. Cell pellets were stored at -20°C until DNA analysis.

### DNA Extraction and qPCR Procedure

Genomic DNA was extracted using the MasterPure^TM^ complete DNA and RNA Purification Kit (Epicentre, Madison, United States), according to the manufacturer’s indications. The quality and concentration of DNA extracts were determined by spectrophotometric measurement at 260/280 and 260/230 nm using a NanoDrop^®^ ND-1000 UV–Vis spectrophotometer (Thermo Fisher Scientific, Inc., Waltham, MA, United States). Quantitative real-time PCR and data analysis were performed using an ABI 7500 Sequence Detection System (ABI, Applied Biosystems, Madrid, Spain). For *L. monocytogenes*, the primer and probe concentrations as well as the cycling parameters and conditions for reactive quantification were as previously reported (Rodriguez-Lázaro et al., 2004). In the case of total bacteria, prime concentrations, cycling parameters, and amplification and detection conditions were as previously described (Truchado et al., 2019). The limit of detection (LOD) was based on the cycle threshold (Ct) value of the last detectable standard.

The samples with Ct values higher than LOQ were classified as non-determined, while Ct values lower than LOQ were classified as positive. LOD was determined to be Ct = 37 (23 cfu per reaction) for *L. monocytogenes* and Ct = 34 (85 cfu per reaction) for total bacteria.

### Statistical Analysis

All experiments were performed at least in duplicate. Calculation and graphical representation of the median and interquartile range (IQR) of Ct values were performed using Sigma Plot 13 Systat Software, Inc. (Addlink Software Scientific, S.L. Barcelona, Spain). Total bacteria levels evaluated by plate count and molecular techniques were log10 transformed. IBM SPSS Statistics 25 was used for statistical analyses. Except when stated otherwise, *P* values below 0.05 were considered statistically significant. Shapiro–Wilk test was performed to assess the normality of the data (*P* > 0.05). Mann–Whitney *U* test was used to examine the differences among treatments.

## Results and Discussion

### Viability of *L. monocytogenes* Cells Using Flow Cytometry

**FIGURE 1:**
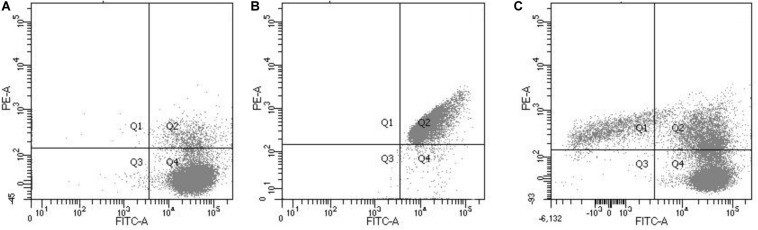
Flow cytometric analysis showing live *L. monocytogenes* cells stained with green fluorescent SYTO 9 **(A)**, dead *L. monocytogenes* cells (isopropanol treated) stained with red fluorescent PI **(B)** and **(C)** a mix culture of live and dead *L. monocytogenes* cells. The Q1 quadrant corresponds with the cells stained with propidium iodine red fluorescent signal (FL2 at 630 nm; dead cells). The green fluorescent signal (FL1 at 520 nm) of the Q4 corresponds with the live cells. The Q2 quadrant corresponds to the double-stained cells (membrane-compromised cells or VBNC) and the Q3 with the non-stained cells.

Flow cytometry was used to determine the proportions of *L. monocytogenes* cells at different states (dead, viable, and intermediate) in untreated and chlorine-treated PWW. Flow cytometry analysis allows the detection of VBNC cells that appear as double-stained cells, indicating that they are still alive but their cell membranes are compromised (Stiefel et al., 2015). This double staining allowed us to establish clearly the population of VBNC cells as shown in Figure 1, in which the potential VBNC cells can be visualized in the Q2 quadrant.

To compare the results obtained by the flow cytometry method with the plate count method, the values obtained by the plate count method were transformed into a percentage. As expected, 100% of culturable cells were obtained for the untreated PWW samples while in the treated PWW samples, it was estimated that 99.9% of the treated cells were dead and not able to grow in the culture medium. However, the flow cytometry methodology estimated that only 65.7% of the cells in the treated PWW were dead, 6.9% were VBNC, and 27.4% of bacteria were completely viable (Table 2). These results could indicate that by the flow cytometry method, bacteria that are not viable are estimated as cultivable. Our results are similar to those obtained by Nocker et al. (2016) with pure cultures of *E. coli*, in which a loss of cultivability of the bacteria with the use of chlorine was detected before any damage in their cell membrane was shown. This is probably because other cellular components were affected by chlorine besides the membrane, such as proteins and lipids (Ersoy et al., 2019), which already make the cells non-viable even if their membranes were not affected. When the two methodologies were compared, the percentage of viable detected cells was higher in the flow cytometry method, which could indicate that flow cytometry could overestimate the percentage of viable cells. For the use of the flow cytometry method to determine VBNC cells, it could be necessary to use other markers of cell damage (DNA, proteins, and lipids) than the membrane. Nocker et al. (2016) observed that in samples of drinking water from a water treatment plant, there were cultivable bacteria that appeared as dead or VBNC by the flow cytometry method, in disagreement with our results. As these authors indicated, this fact could be due to the more heterogeneous natural microbiota with different susceptibilities to the disinfectant in water samples. In our study, due probably to the differences in the natural microbiota from lettuce, the results differed.

**TABLE 2:** Percentage of viable, viable but non-culturable (VBNC), and dead cells of a six-strain cocktail of *Listeria monocytogenes* untreated and treated with chlorine (10 mg/L) determined by plate count and flow cytometry methods.

		Physiological Cell Stage (%)		
Methodology	Treatments	Viable	VBNC	Dead
Plate Count	Untreated	100.0		0.0
	Treated	0.0		99.9
Flow cytometry	Untreated	89.6 ± 2.3	7.8 ± 1.0	2.6 ± 1.2
	Treated	27.4 ± 3.3	6.9 ± 0.1	65.7 ± 3.3

Taking into account the complex composition of the PWW, with high organic matter content and interfering compounds, the fluorescent dyes were not able to differentiate among the physiological stage of the different bacteria species. Based on the results obtained, the flow cytometry was not a suitable methodology to distinguish between viable and dead cells in PWW. Several studies have suggested the use of flow cytometry as an accurate analytical tool to determine the viability against sanitizers of the foodborne pathogenic bacteria (Nocker et al., 2016; Ersoy et al., 2019). However, these studies were mostly performed using tap water or pure cultures, without considering the complex composition of PWW in industrial settings (Gombas et al., 2017).

### Viability of *L. monocytogenes* Cells Using v-qPCR Techniques Combined With PMAxx

**FIGURE 2:**
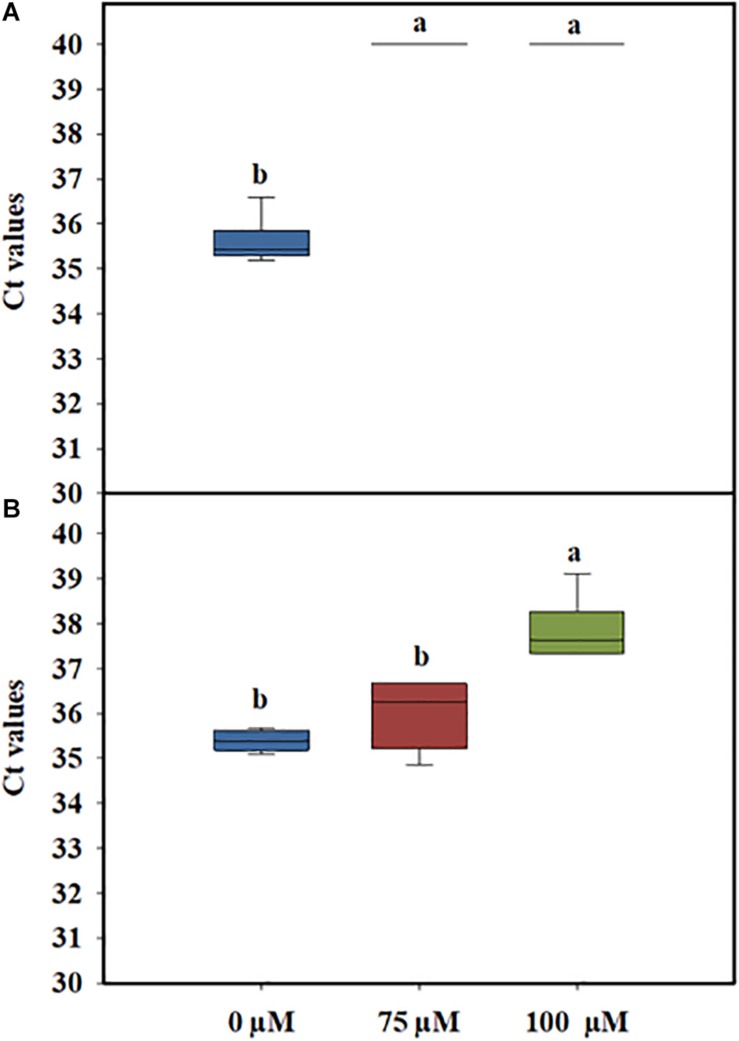
Cycle threshold (Ct) values obtained by PMAxx-qPCR at different concentrations with DNA extracted from live **(A)** or heat-killed **(B)** cells of a six-strain cocktail of *Listeria monocytogenes* inoculated in process wash water. The bottom and top of the boxes represent the quartiles (25th and 75th percentiles, respectively), with the line inside the box representing the median. Whiskers show the highest values (defined as values more than 3/2 times the corresponding quartile). Different letters indicate significant difference at *P* < 0.05.

In general, PMA concentrations of 50 μM have been reported to be the optimal concentration for the efficient differentiation between live and dead cells without affecting the viability of *L. monocytogenes* cells (Kragh et al., 2020). However, when complex matrixes, such as PWW, are used, the concentration of PMAxx needs to be optimized to avoid any impact on cell viability. Preliminary experiments performed to determine the optimal concentration of PMAxx showed that when 50 μM of PMAxx was added to PWW, the PCR signal of dead cells was not fully discriminative (data not shown). One reason could be the presence of organic matter in the PWW, which might interfere with the photoreactive DNA dye, reducing its ability to bind to the DNA of the dead cells. Based on these results, two higher concentrations of PMAxx (75 and 100 μM) were tested. The quantitative PCR Ct values of heat-killed *L. monocytogenes* treated with 75 and 100 μM PMAxx concentrations were not significantly different (Figure 2A). However, a toxicity effect was observed in the live cells when a higher concentration of the dye was used (Figure 2B). Therefore, to avoid any impact on the viability of the *L. monocytogenes* cells, the lowest concentration (75 μM) of PMAxx was selected for further analysis.

Several studies have focused on the optimization of methodologies able to discriminate between live and dead cells of pathogenic bacteria such as *Xylella fastidiosa*, *Vibrio parahaemolyticus*, and *Clavibacter michiganensis* ssp. *michiganensis* in complex matrixes such as plants, shrimp, and seed tomato, respectively (Han et al., 2018; Cao et al., 2019b; Sicard et al., 2019). Most of these studies evidence the good discriminative effect of PMAxx when compared to PMA between live and dead cells in complex matrixes. However, as far as we know, differentiation between live and dead *L. monocytogenes* in PWW has not been accomplished.

### v-qPCR Combined With PMAxx + EMA

**FIGURE 3:**
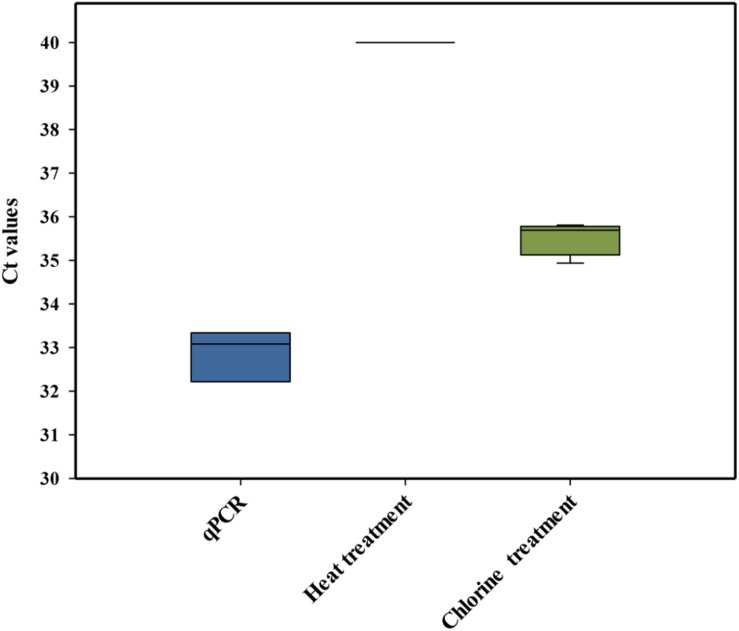
Cycle threshold (Ct) values obtained by PMAxx-qPCR with DNA extracted from cells of a six-strain cocktail of *Listeria monocytogenes* inoculated in heat-treated or chlorine-treated process wash water. The box plot represents three replicates of two independent assays (*n* = 6). The bottom and top of the boxes represent the quartiles (25th and 75th percentiles, respectively), with the line inside the box representing the median. Whiskers show the highest values (defined as values more than 3/2 times the corresponding quartile).

Laboratory-scale experiments were performed to study the mode of action by which chlorine killed *L. monocytogenes* cells and whether or not the membrane integrity of the cells was affected. It has been accepted that chlorine and other chemical sanitizers usually inactivate the bacterial cell by the disruption of the cytoplasmic membrane (Venkobachar et al., 1997; Nocker et al., 2007). However, it is essential to determine the suitability of PMAxx to differentiate between live and dead cells when chlorine treatments are applied. In these experiments, PCR amplification of dead *L. monocytogenes* cells killed by heat treatment was compared with those killed by chlorine. The initial conditions used for this comparison were those previously recommended to discriminate dead *L. monocytogenes*, including 30 min of dye incubation at 40°C (Nkuipou-Kenfack et al., 2013). As expected, *L. monocytogenes* DNA activated with PMAxx (75 μM) and isolated from heat-treated *L. monocytogenes* did not show amplification in the qPCR. However, the PCR signal of *L. monocytogenes* DNA activated with PMAxx (75 μM) obtained from chorine-treated cells was not completely inactivated, showing a Ct value below the LOD (Figure 3). Our results agree with a previous study that observed that PMA-qPCR assay (PMA at 50 μM for 20 min of incubation at 37°C) did not reduce the signal of chlorine-killed cells of *E. coli* O:157 H7 (10^4^ cfu/ml) artificially inoculated in drinking water (Cao et al., 2019a). Based on these results, new experiments were performed under different conditions of time/temperature for the incubation of the DNA with the dye. However, none of the tested combinations improved previous results obtained (data not shown).

The results indicated that there was an overestimation of the live cells because some dead cells could still be quantified (false-positive results) after the chlorine treatment, mostly due to the presence of an intact membrane. A similar conclusion was also achieved by Virto et al. (2005) who observed that membrane damage is not the only event involved in the inactivation of bacteria by chlorine. These authors also highlighted that the presence of organic matter could play an important role in the accessibility of chlorine to the bacteria, influencing their resistance. Based on these findings, the PMAxx-qPCR method was not considered a suitable approach to differentiate between live and dead *L. monocytogenes* cells present in PWW treated with chlorine.

**FIGURE 4:**
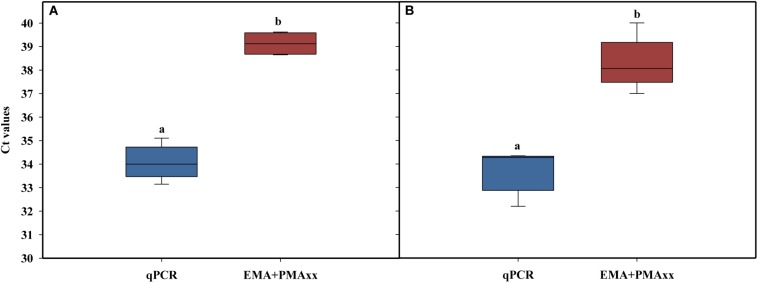
Effect of EMA + PMAxx incubation time on cycle threshold values obtained in qPCR with DNA extracted from dead cells of a six-strain cocktail of *Listeria monocytogenes* inoculated in heat-treated or chlorine-treated process wash water. Time of incubation was 40 min **(A)** and 60 min **(B)**. Different letters denote significant differences (*P* < 0.05). The box plot represents three replicates of two independent assays (*n* = 6). The bottom and top of the boxes represent the quartiles (25th and 75th percentiles, respectively), with the line inside the box representing the median. Whiskers show the highest values excluding outliers, and dots represent outliers (defined as values more than 3/2 times the corresponding quartile).

The alternative method studied was EMA combined with PMAxx, to detect both membrane integrity and active metabolism (Codony, 2014; Codony et al., 2015; Agustí et al., 2017). Several authors have suggested that cell viability should include cells that have intact, functional, and active membranes (Codony et al., 2015). Some studies related to membrane integrity reported that the combination of EMA and PMA reduced the DNA signal from dead cells (intact and damage membrane) and live cells with inactive membranes (Codony et al., 2015). This method is based on the EMA properties, which accumulate in dormant cells that lack the metabolic ability to offset its uptake using active mechanisms such as efflux pumps. Concentrations of 75 μM of PMAxx and 10 μM of EMA were used followed by incubation at 40°C at two incubation times (40 and 60 min) and a 15-min light exposure. The results showed that, independently of incubation time, the combination of the two photoreactive dyes (PMAxx and EMA) reduced the amplification of dead cells after chlorine treatment above the LOD (Ct value > 37; Figure 4). When the two incubation times were compared, a slight increase in the Ct values was observed when 60 min was applied versus 40 min (Ct value = 39 and 37, respectively). However, it should be considered that a long incubation time might have a negative effect on the viability of *L. monocytogenes* cells inoculated in PWW. The differences between the levels of *L. monocytogenes* enumerated by qPCR and EMA + PMAxx-qPCR are shown in Table 3. Based on these results, an incubation time of 40 min was selected. The latest studies demonstrated that the commercial PEMAX reagent (a new commercial reagent that combines both EMA and PMA) is suitable in food matrixes and complex environmental samples, avoiding the overestimation of the most common methodologies (Agustí et al., 2017; Daranas et al., 2018).

**TABLE 3:** *Listeria monocytogenes* counts inoculated in process wash water detected by qPCR combined or not with EMA + PMAxx at two incubation times.

Time of incubation (min)	*L. monocytogenes* (log cfu/ml)	
	qPCR	EMA + PMAxx-qPCR
40	4.14 ± 0.10a	3.83 ± 0.11a
60	4.34 ± 0.02a	3.29 ± 0.06b

*Values represent the mean ± standard deviations for three independent replicates.*

### Validation of the Detection and Quantification Method for VBNC Cells

The detection method selected, EMA + PMAxx-qPCR, was validated in PWW samples obtained in an industrial processing line where shredded lettuce was washed with chlorine. Based on the results obtained, no significant changes in the concentration of free chlorine and pH were observed along with the sampling interval that was able to control the accumulation of bacteria in the PWW, maintaining a total count below 3 log units (Table 1). Similar results have been previously described in several industrial washing lines (López-Gálvez et al., 2019; Tudela et al., 2019).

**FIGURE 5:**
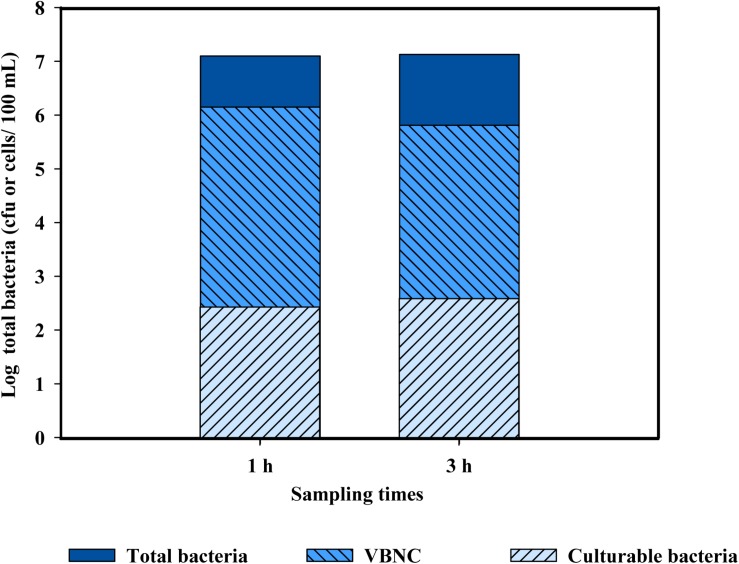
Populations of total bacteria (log cfu or cells/100 ml) in chlorine-treated process wash water used for washing shredded lettuce after 2 and 3 h from the beginning of the washing, corresponding to time 1 and time 3, respectively. Levels of culturable bacteria were obtained by plate count, levels of total bacterial by qPCR, and viable bacteria by EMA + PMAxx-qPCR. Levels of VBNC were calculated by the differences between viable and culturable bacteria and dead cells by the differences between total bacteria and viable bacteria.

When the levels of total bacteria present in PWW were quantified, the results obtained by cultivation-based methods were very different than those based by molecular-based techniques (both qPCR and EMA + PMAxx-qPCR). Several studies have reported that the use of plate count methods leads to an underestimation of the total bacterial levels in environmental samples, mostly due to the presence of cells in the VBNC state (Rastogi et al., 2010; Truchado et al., 2019). The results obtained indicate that chlorine (10 mg/l) induced bacteria into a VBNC state (Figure 5). Only slight differences were observed between the two sampling points (1 and 3 h). These results agree with those of Highmore et al. (2018) who demonstrated that the use of chlorine induced cells of *Salmonella enteritidis* and *L. monocytogenes* into a VBNC state. However, the conditions applied in this referenced study (Highmore et al., 2018) did not represent real conditions of a fresh-cut processing line, which include the presence of high concentrations of organic matter and background microbiota in the PWW. This is the first study showing the induction of the VBNC state of bacteria cells present in PWW when water was disinfected with chlorine in industrial settings.

## Conclusion

The v-qPCR combined with the two DNA amplificatory inhibitors (EMA and PMAxx) represents the optimization technique for the detection and quantification of cells in the VBNC state. The use of these photoreactive DNA–dye combinations (EMA and PMAxx) yields a more accurate estimation of the *L. monocytogenes* viable cells present in PWW than other methodologies tested, such as flow cytometry and PMAxx-qPCR. Concentrations of 10 μM EMA and 75 μM PMAxx and incubation at 40°C for 40 min followed by a 15-min light exposure allowed the inactivation of most of the dead cells. The validation of the method in an industrial setting showed that the methodology optimized could significantly distinguish dead and VBNC cells in PWW treated with chlorine. This method can be considered as a rapid and reliable one recommended for the detection of VBNC cells in complex water matrixes such as those of the food industry. However, complete discrimination between dead and VBNC cells was not achieved, which led to a slight overestimation on the percentage of VBNC cells in the PWW, mostly due to the complex composition of this type of wash water. The verification of the methodology optimized with different sanitizers is worthy of being investigated.

## Data Availability Statement

The raw data supporting the conclusions of this article will be made available by the authors, without undue reservation, to any qualified researcher.

## Author Contributions

PT, MG, and AA designed this experiment. PT conducted the molecular lab work and data analysis. ML conducted the cytometry flow lab work and data analysis. PT drafted the manuscript under the advisement of AA. All authors met to develop methods used for conducting the study and read the draft and provided feedback.

## Conflict of Interest

The authors declare that the research was conducted in the absence of any commercial or financial relationships that could be construed as a potential conflict of interest.
